# Considering Commercial Vessels as Potential Vectors of Stony Coral Tissue Loss Disease

**DOI:** 10.3389/fmars.2021.709764

**Published:** 2021-09-22

**Authors:** Nicholas A. Rosenau, Sarah Gignoux-Wolfsohn, Richard A. Everett, A. Whitman Miller, Mark S. Minton, Gregory M. Ruiz

**Affiliations:** 1 Ocean and Coastal Management Branch, Office of Wetlands, Oceans, and Watersheds, United States Environmental Protection Agency, Washington, DC, United States,; 2 Smithsonian Environmental Research Center, Edgewater, MD, United States,; 3 United States Coast Guard, Office of Operating and Environmental Standards, Washington, DC, United States

**Keywords:** ballast water, vessel discharge regulations, stony coral tissue loss disease, Caribbean, vector of stony coral tissue loss disease, biofouling

## Abstract

Stony coral tissue loss disease (SCTLD) is a troubling new disease that is spreading rapidly across the greater Caribbean region, but the etiological agent(s) and the mechanisms(s) of spread are both unknown. First detected off the coast of Miami, Florida, major ocean currents alone do not explain the pattern of spread, with outbreaks occurring across geographically disjunct and distant locations. This has raised concerns by researchers and resource managers that commercial vessels may contribute as vectors to spread of the disease. Despite existing regulatory and management strategies intended to limit coastal marine invasion risks, the efficacy of these measures is still unresolved for ship-borne microorganisms, and disease transport *via* ballast water and hull biofouling are under examination given the high ship traffic in the region. Here, to help inform the discussion of ships as possible vectors of SCTLD, we provide an overview of the current state of knowledge about ships and their potential to transfer organisms in the greater Caribbean, focusing in particular on ballast water, and outline a set of recommendations for future research.

## INTRODUCTION

**FIGURE 1 |:**
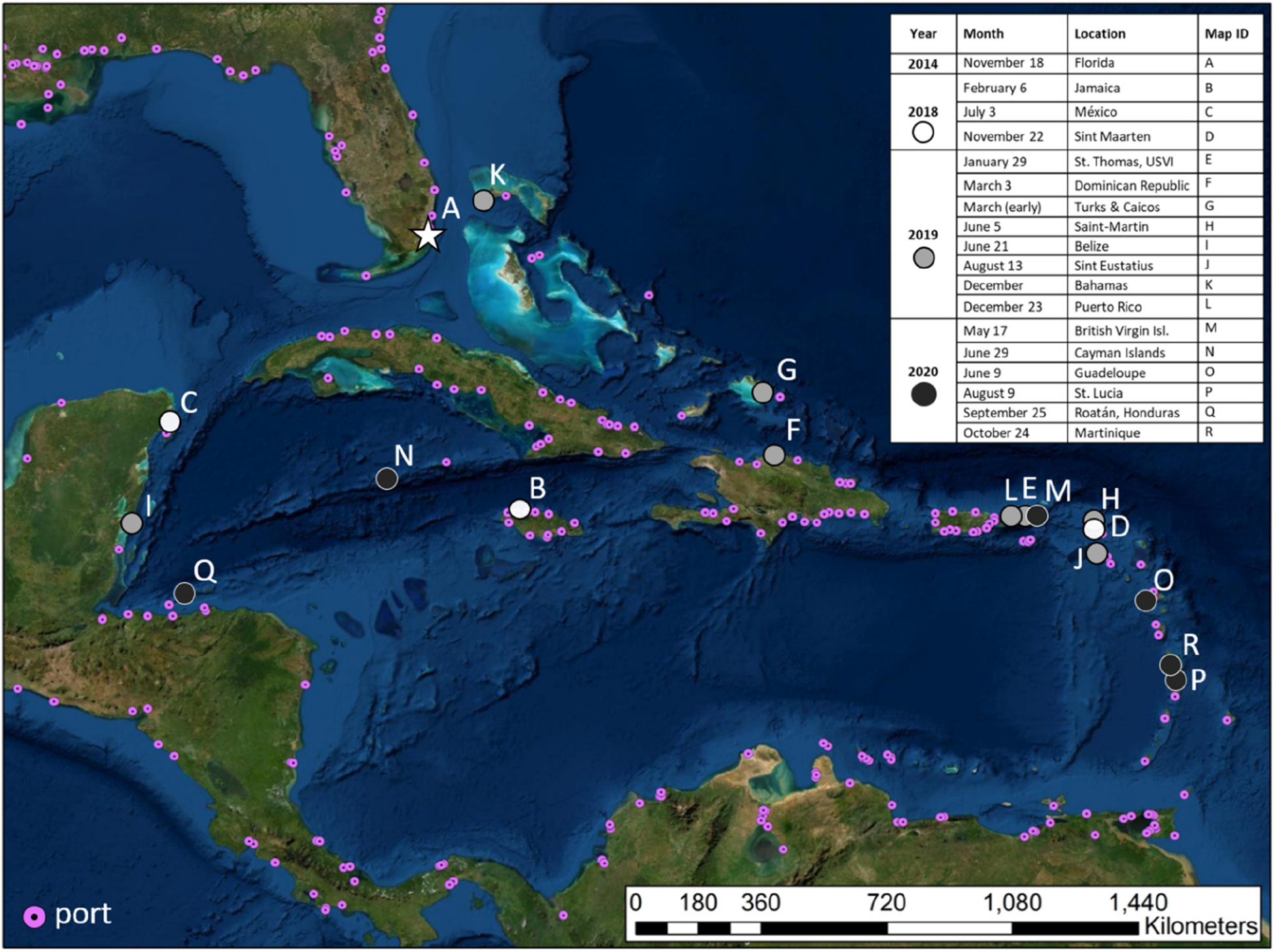
Spatial distribution of stony coral tissue loss disease across the Caribbean through time. Map modified from Roth et al. (2020).

Stony coral tissue loss disease (SCTLD), a lethal coral disease first reported off the coast of Miami-Dade County, Florida in September 2014 (Precht et al., 2016; Walton et al., 2018), has spread quickly and as of July 2021 has been confirmed in 15 countries/territories throughout the greater Caribbean region (Figure 1; Kramer et al., 2019). Communities depend on coral reef ecosystems for fisheries, coastal protection, and income from tourism. The quick and persistent spread of SCTLD, and still-mysterious mechanism(s) of transport, remain a source of great concern. Because of the unprecedented number of coral species affected (~20) for coral diseases, there are also concerns that Pacific corals may be susceptible to the disease, prompting alarm about pathogen movement beyond the Caribbean.

As with many other coral tissue loss diseases (Roder et al., 2014; Gignoux-Wolfsohn and Vollmer, 2015), the etiological agent(s) of SCTLD has not been identified. Transmission can occur through water and coral-to-coral contact, and is reduced by antibiotics (Aeby et al., 2019; Muller et al., 2020), indicating biological agents. Ocean currents have been posited as a mode of spread along the Florida Reef Tract (Aeby et al., 2019; Muller et al., 2020) but are not sufficient to explain the spread across the greater Caribbean, motivating the exploration of other modes of transmission including human-mediated transport. One potential mechanism receiving considerable attention is unintended vessel-related transport. While no definitive connection exists between any transport mode and coral disease outbreaks, it is reasonable to consider vessels as potential vectors of pathogens. As the timing and pattern of SCTLD spread through the Caribbean has been better documented than other Caribbean coral disease to date, this disease provides an unprecedented opportunity to examine spatial patterns of coral disease movement.

Vessels are known to be a significant global vector for the spread of aquatic non-native species in coastal waters (Carlton, 1985; Ruiz et al., 2000a; Hewitt et al., 2009; Bailey et al., 2020). A taxonomically diverse biota is transported on or in vessels *via* ballast water (BW), biofouling (BF) of BW tank walls and on exterior submerged surfaces, bilges, fish holds, fishing gear, and anchor chains (Fofonoff et al., 2003). The role of vessels in transporting organisms varies as a function of vessel size, design, operations, and transit distances/routes. Commercial cargo vessels, as well as recreational and fishing vessels, can all transport non-native organisms (Ashton et al., 2014; Zabin et al., 2014; Ulman et al., 2019).

Compared to macrobiota, our understanding of microbial transport *via* vessels remains limited, as most research to date has focused on movement of large and easily identifiable species, especially invertebrates and phytoplankton (Pagenkopp Lohan et al., 2020). An abundant and diverse assemblage of microorganisms, including pathogens and parasites, have been found in BW, and in sediments and surface biofilms of BW tank (Ruiz et al., 2000b; Drake et al., 2007; Pagenkopp Lohan et al., 2016). However, the extent to which invasions and disease epizootics result from microorganism transfers remains poorly resolved (Pagenkopp Lohan et al., 2020). Further, the transport of microorganisms in vessels’ external BF has been especially poorly studied.

Here, we share our perspective on large commercial vessels (hereafter “ships”), which transport cargo and people, as potential vectors for pathogens as a possible cause SCTLD, focusing particularly on BW, and we do not explore other means by which ships can transport organisms or recreational vessels. We provide a brief overview of BW and relevant United States and international regulations on BW management. We then summarize current efforts to evaluate ships as a potential vector for SCTLD. Finally, we provide recommendations for future research to address key knowledge gaps. We intend this perspective piece to inform research and management strategies on the potential for ship-mediated spread of SCTLD.

## BALLAST WATER

### Mechanism of Organism Transport

Ballast water is fresh or seawater, not wastewater, held in tanks and holds of ships to manage draft, trim, and stability. BW is taken up both in ports and while in transit, and contains a wide diversity of organisms from the water column and shallow benthic sediments (Ruiz et al., 2000b; Drake et al., 2001, 2007). Rarely, ships obtain BW from municipal water sources (National Ballast Information Clearinghouse [NBIC], 2021). Ships take on and discharge BW at various locations (e.g., in-harbor while undergoing cargo operations, at anchorage, at a lightering area outside of harbor where cargo is transferred between ships, or en route between port locations) resulting in large-scale species transfer (National Research Council, 2011). For context, in 2018, ships reported discharges of 365.2 million m^3^ of BW into United States waters (National Ballast Information Clearinghouse [NBIC], 2021).

While ships are in the water, BW tanks are never completely empty, due to limitations of the pumping systems. Consequently, there can be significant amounts of residual BW and sediment in “empty” tanks (Bailey et al., 2003), and biota from many locations may accumulate in BW tanks over time. Thus, the “source” of BW and its associated organisms often cannot be attributed exclusively to the last departure place of the ship, because BW may be taken up and commingled across a variety of locations.

### Transport of Microbes in Ballast Tanks

A diverse assemblage of microbes, including bacteria and viruses, is carried in BW (Ruiz et al., 2000b; Drake et al., 2001, 2007; Aguirre-Macedo et al., 2008; Kim et al., 2015; Pagenkopp Lohan et al., 2016, 2017; Lymperopoulou and Dobbs, 2017; Hwang et al., 2018). Additionally, bacteria are also present in biofilms on internal surfaces (Drake et al., 2005, 2007) and in sediments at the bottom of BW tanks (Drake et al., 2007). Surface- and sediment-associated microbes may be shed within BW tanks into the resident BW *via* biological and physical processes, and subsequently discharged with the BW (Drake et al., 2005).

### Ballast Water Management

Following several species invasions with large ecological and social impacts in the 1980s (Mills et al., 1993; Oliveira, 2007), BW management regulations have advanced within individual countries and internationally (National Research Council, 1996, 2011). In the United States, a series of laws and regulations mandating BW management (Table 1) has resulted in a stepwise reduction of coastal organism concentrations discharged in BW over the past 35 years (Minton et al., 2005; Albert et al., 2013; Carney et al., 2017).

Under United States Coast Guard (USCG) regulations, all ships that are capable of carrying BW and bound for ports or places in the United States are required to submit a BW management report to the National Ballast Information Clearinghouse (NBIC^1^) for each arrival. These reports identify, for every tank of BW discharged, the location and date of all BW sources, discharges, and BW management methods applied.

### Ballast Water Exchange

Ballast water exchange (BWE) was originally used by ship masters to reduce the amount of sediment buildup in ballast tanks. This practice was then adopted to reduce transport of coastal organisms. Initial guidelines were disseminated by governments and the International Maritime Organization (IMO) (Locke et al., 1991, p. 47; International Maritime Organization, 1993, 1997). Under USCG rules, a ship conducting BWE flushes out coastal BW (and organisms therein) from its tanks at least 200 nautical miles (Nm) from any land mass.

Ballast water exchange is conducted in the open ocean either by continuously pumping three full tank volumes of water through each tank or by pumping out the original water until the pump loses suction, and then refilling. BWE replaces coastal BW or organisms with open ocean water, reducing the concentration of coastal organisms in BW tanks but not eliminating them (Minton et al., 2005; Ruiz and Reid, 2007; National Research Council, 2011). Such reductions in the concentration of coastal organisms are expected to lower the likelihood of invasions, because most BW-associated invasions are reported nearshore (Ruiz et al., 2000a,b) and invasion probability scales with the abundance of organisms released (National Research Council, 2011).

Similar regulations have been established internationally by the IMO (International Maritime Organization, 2004) under the International Convention for Management of Ships’ Ballast Water and Sediments (2004) (hereafter “the Convention”). Specifically, the Convention requires that ships conduct BWE at least 50 Nm from shore. Ships of participating countries (not including the United States) must abide by the Convention’s requirements wherever they operate. Thus, ships of parties to the Convention transiting to non-United States places in the greater Caribbean should be conducting BWE > 50 Nm from shore whenever possible, if managing BW using BWE. Both United States and Convention regulations exempt ships if conducting BWE is deemed unsafe due to ship design or sea conditions, and if BWE would require diversion or delay of voyages. Importantly, USCG requires that exempted ships discharge as little BW as is operationally practicable.

### Ballast Water Management Systems

To further reduce the number of organisms and invasion risk beyond BWE, USCG, and Convention regulations phase in concentration-based limits on organisms in BW discharge. The numeric limits are the same in the two standards: fewer than 10 organisms with a minimum dimension ≥50 μm per m^3^; fewer than 10 organisms between 50 and 10 μm per mL; and fewer than 1 colony-forming unit (CFU) of *Vibrio cholerae* (serotypes O1 and O139), 250 CFU of *Escherichia coli*, and 100 CFU of enterococci per 100 mL. These limits are largely achieved through use of ballast water management systems (BWMS). Treatment with a BWMS is more effective for meeting concentration-based discharge standards than conducting BWE (Minton et al., 2005; Carney et al., 2017) and does not have the same operational constraints as BWE (i.e., distance from shore or safety).

Ballast water management systems are generally comprised of several components, including one or more designed to kill, remove, or render non-viable organisms in BW. Filters are often incorporated to remove larger organisms prior to chemical or physical disinfection. Various chlorine-based oxidants and ultraviolet radiation are the most widely employed disinfecting processes. At the time of writing, the USCG has approved 43 BWMS^2^ and accepted, for temporary use, over 100 BWMS approved under the Convention.

Besides the human pathogens mentioned above, United States and Convention regulations place no limits on general or total organisms <10 μm. While microbes would presumably be reduced in concentration by BWMS, research is needed on the effects of BWMS on potentially pathogenic microbes. Attention should be paid to marine microbes in a viable but non-culturable state, as current culture-based standards may not reflect actual presence of microbes. Furthermore, concentration limits may not be effective against asexually-reproducing coral pathogens depending on infectious dose and favorability for pathogen reproduction at discharge locations.

## BALLAST WATER AND SCTLD

Appearances of SCTLD at geographically distant locations, and first reports near ports (Atlantic Gulf Rapid Reef Assessment [AGGRA], 2020), has invited questions about BW as a possible vector. In 2019, the United States Coral Reef Task Force^3^ asked the United States Environmental Protection Agency (EPA) to evaluate BW as a potential SCTLD vector. Subsequently, EPA established a collaboration with USCG and the Smithsonian Environmental Research Center to evaluate BW discharges and shipping patterns within the context of SCTLD spread. Additionally, the Naval Research Laboratory and the National Oceanic and Atmospheric Administration (NOAA) Atlantic Oceanographic and Meteorological Laboratory are collaboratively investigating the degree to which SCTLD-exposed water can remain infectious, and the efficacy of UV treatment.

### Ballast Water Movement and SCTLD Patterns

United States BW management data and Caribbean-wide SCTLD report information are being used in projects to evaluate BW as a potential vector for SCTLD. However, such analyses are correlative and often do not yet consider other possible mechanisms or interactions. Correlations between disease incidences and ship movements or BW discharge are useful, but not sufficient to definitively identify ships as vectors. Importantly, given the extensive maritime traffic in the Caribbean, including both ships and recreational vessels, we urge caution in drawing conclusions based on apparent correlations, in the absence of experiments demonstrating causation.

A preliminary analysis in 2019, including >36,000 BW discharge and management records in the NBIC, spanning Sep 7, 2013–Aug 6, 2020, detected no clear relationship between BW discharges and SCTLD occurrence in United States waters, since sites with SCTLD varied in BW discharge histories, including some with no reported discharge.

Following these initial results, on September 6, 2019, at the request of NOAA, USCG, in collaboration with EPA, issued a Marine Safety Information Bulletin (MSIB; United States Coast Guard, 2019), advising mariners of the SCTLD outbreak, reminding them of BW management regulations, and recommending voluntary BWE practices to reduce possible spread of the disease. Over the 12 months following the MSIB, the number of ships discharging BW that is unmanaged (i.e., without measures to reduce the concentration of organisms prior to discharge) within 12 Nm of the United States shore on a monthly basis was lower than the average number doing so for the six years prior to the MSIB. However, there was not a compensatory increase in the number or proportion of BWE events beyond 50 Nm, as might be expected if ships were following the recommendations in the MSIB, making it impossible to attribute this decrease solely to the MSIB. Instead, the increase in ships using BWMS rather than BWE, combined with the decline in ship arrivals due to the COVID-19 pandemic, may account for some or all of the observed decrease in discharge of unmanaged BW (Everett et al., 2021).

The MSIB, as a rapid communication, avoided the significant administrative timelines associated with new regulations; however, it has not appreciably decreased the potential risk of transporting coral pathogens to United States locations in the greater Caribbean through the discharge of unmanaged BW. The increasing use of BWMS to treat BW prior to discharge is expected to significantly reduce the risks of pathogen transport, but efficacy of treatment methods against organisms causing SCTLD needs to be directly tested. If BW is identified as a contributor to SCTLD spread, mitigation may need to include approved treatment systems, and depending on the efficacy of such, new regulations for BW management.

## CONSIDERATION AND FUTURE RESEARCH

Stony coral tissue loss disease continues to spread across the greater Caribbean and there are indications that Pacific corals may also be susceptible to the disease (Valerie Paul, personal communication), prompting concern about pathogen movement *via* ships to other geographic regions. It is therefore imperative that we understand whether the disease can be transferred *via* ship BW and/or BF, and under what circumstances. While SCTLD warrants such attention due to its significant impacts, it also provides an important model to understand the broader risk associated with microorganism transfers.

While ships clearly have the potential to spread pathogens, understanding the likelihood of ship-mediated spread of SCTLD remains challenging given the unknown etiology of the disease. While successful treatment with antibiotics suggests the pathogen(s) may be bacterial (Aeby et al., 2019; Neely et al., 2020), we cannot rule out a possible role of free-living or bacteria-infecting viruses, other microorganisms (Rosales et al., 2020), or water quality (Miller et al., 2016; Cunning et al., 2019; Rosales et al., 2019). Additionally, the potential that SCTLD is caused by a consortium of pathogens complicates development of targeted tests or treatments. Experiments are needed to evaluate the effects of voyage characteristics on transmissibility of SCTLD and treatment with BWMS. Current treatment experiments focus on UV irradiation; however, other disinfection processes widely used in BWMS should also be investigated. Ultimately, identifying the pathogen(s) will greatly aid effective prevention.

Concerns about ship-borne transport of SCTLD have focused on free-living pathogens in BW, but pathogens could also be spread as biofilms on internal or external surfaces, or within animal hosts. Zooplankton, commonly transported in BW, can act as a vector for other coral diseases (Certner et al., 2017). In addition, animals such as snails (Gignoux-Wolfsohn et al., 2012; Nicolet et al., 2013) and fireworms (Sussman et al., 2003) have been shown to harbor and transmit coral pathogens. Understanding the ecology of SCTLD, including potential biotic reservoirs and vectors in BW and BF, is crucial to controlling disease spread *via* ships. As with BW, the flux of BF organisms is potentially large, given the cumulative surface area that arrives from other bioregions to United States waters alone (Miller et al., 2018), and the still limited requirements for BF management (Davidson et al., 2016, 2018; Tamburri et al., 2020; Georgiades et al., 2021). Furthermore, numerous smaller vessels (e.g., recreational, fishing, and barges) also may transfer biofouling organisms across the greater Caribbean region. Currently, we know of no broadly applicable national or international biosecurity requirements regarding BF in the greater Caribbean region.

There is a demonstrated link between temperature and SCTLD, with lesion progression slowing during periods of thermal stress (Meiling et al., 2020). Investigation of correlations between SCTLD outbreaks and ships should consider possible interactions with environmental conditions, especially in the context of climate and habitat changes. Next steps should include a statistically-rigorous spatio-temporal analysis of the relationships between SCTLD occurrence, currents, environmental covariates (temperature, salinity, and turbidity), and ship/BW flux. This analysis should include the alternate hypothesis that spatio-temporal patterns of outbreaks are not due to pathogen transport, but rather a trigger (e.g., environmental change) inducing pathogenicity. BW information in the NBIC provides a rich data source on ships and BW discharges in United States waters. Similar information for non-United States ports in the greater Caribbean region would be a valuable addition to this analysis.

Importantly, safety and route exemptions from requirements for BWE (considered necessary for ship safety and smooth maritime transportation) mean that a significant proportion of BW flux among ports within the region will remain unmanaged until existing ships operating in the region reach their United States and Convention discharge standard compliance dates. Additional interim best management practices, and/or emergency measures, to reduce the transport of SCTLD by these otherwise exempted ships should be considered.

While efforts are underway to understand the potential pathways by which SCTLD spreads geographically, no definitive connection between any transport mode and disease outbreaks has yet been verified. It is reasonable to recognize ships as potential vectors for transmission of SCTLD, *via* BW and BF, at a large geographic scale; however, their roles in the dynamics of this disease are still not clear. We recommend continued focused research on these possible mechanisms of spread, in concert with ongoing efforts to understand disease etiology and ecology. We also encourage collaborations that bring together experts in ships/shipping, water disinfection, and coral disease.

## Supplementary Material

Sup.

## Figures and Tables

**TABLE 1 |:** Timeline of significant legislation and regulation in the United States regarding ballast water management.

Year	Author	Publication	Action
1990	USC	Law—Non-indigenous Aquatic Nuisance Prevention and Control Act (NANPCA; 16 U.S.C. 4711 et seq.)	Established USCG regulatory jurisdiction over BW management
1993	USCG	Regulation—Ballast Water Management for Vessels Entering the Great Lakes (58 FR 18330)	Ships required to conduct BWE prior to entry into the Great Lakes
1994	USCG	Regulation—Ballast Water Management for Vessels Entering the Hudson River (59 FR 67632)	Ships required to conduct BWE prior to entry into the Hudson River
1996	USC	Law—National Invasive Species Act (NISA; 16 U.S.C. 4711 et seq.)	USCG required to establish national BWM guidelines and reporting requirements, evaluate industry participation, and report to Congress
1999	USCG	Regulation- Implementation of the National Invasive Species Act of 1996 (64 FR 26672)	Established voluntary BWM guidelines, including BWE; recordkeeping and reporting requirements
2002	USCG	Report to Congress on the Voluntary National Guidelines for Ballast Water Management (USCG-2002-13147 at http://www.regulations.gov)	Reported a finding of low participation by industry and poor reporting. Triggered NISA requirement that voluntary program become mandatory
2004	USCG:	Regulation—Penalties for Non-submission of Ballast Water Management Reports (69 FR 32864)	Instituted penalties for failure to submit required reports and maintain required records
2004	USCG:	Regulation—Mandatory Ballast Water Management Program for United States Waters (69 FR 44952)	Ships required to conduct a mid-ocean BWE, retain BW onboard, or use an approved alternative BWM method
2005	USCG:	Policy—Ballast Water Management for Vessels Entering the Great Lakes that Declare No Ballast Onboard (70 FR 51831)	Established BMP: ships entering the Great Lakes with empty BW tanks should conduct mid-ocean flush of “empty” tanks
2008	USEPA	Vessel General Permit (VGP; 73 FR 79473)	Established BWM requirements for ships under the Clean Water Act (CWA): USCG requirements plus BWE for ships engaged in Pacific nearshore voyages, and mid-ocean flush of “empty” BW tanks prior to entry into Great Lakes
2012	USCG	Standards for Living Organisms in Ships’ Ballast Water Discharged in United States Waters (77 FR 17254)	Ships required to use approved BWMS to meet BW discharge standard, use public water supplies, or discharge to reception facilities; phase out BWE
2013	USEPA	VGP (78 FR 21938)	Applied USCG BW discharge standard to a broader suite of vessels than USCG rule
2018	USC	Vessel Incidental Discharge Act [VIDA; 33 U.S.C. 1322(p)]	Repealed existing USCG and EPA authorizations, effective upon publications of new ship discharge standards by EPA and compliance regulations by USCG

BWM, ballast water management; BWE, ballast water exchange; USC, United States Congress; USCG, United States Coast Guard; United States EPA, United States Environmental Protection Agency; U.S.C, United States Code; FR, Federal Register.

## Data Availability

The original contributions presented in the study are included in the article/supplementary material, further inquiries can be directed to the corresponding author/s.

## Footnotes

1
www.nbic.si.edu

2
https://cgmix.uscg.mil

3
https://www.coralreef.gov
