# Supplementary material for: Considering Commercial Vessels as Potential Vectors of Stony Coral Tissue Loss Disease
Source: Front Mar Sci. Author manuscript; Available in PMC 2022 Jun 8. (PMC9175181; doi:10.3389/fmars.2021.709764)
Supplement: Sup. [file NIHMS1811397-supplement-Sup_.docx]

Perspective: Considering Commercial Vessels as Potential Vectors of Stony Coral Tissue Loss Disease

Nicholas A. Rosenau^1*^, Sarah Gignoux-Wolfsohn^2^, Richard A. Everett^3**^, A. Whitman Miller^2^, Mark S. Minton^2^, Gregory M. Ruiz^2^

^1^U.S. EPA, Office of Wetlands, Oceans and Watersheds, Washington, DC, USA

^2^Smithsonian Environmental Research Center, Edgewater, MD, USA

^3^U.S. Coast Guard, Office of Operating and Environmental Standards, Washington, DC, USA

Correspondence
[rosenau.nicholas@epa.gov](mailto:rosenau.nicholas@epa.gov)

*The views expressed herein are those of the authors and do not necessarily represent the views or policies of the U.S. Environmental Protection Agency

** The views expressed herein are those of the author and are not to be construed as official or reflecting the views of the Commandant or of the U. S. Coast Guard.

Words: 2,997; Figures: 1; Tables: 1

Keywords: ballast water, vessel discharge regulations, stony coral tissue loss disease, Caribbean, vector of stony coral tissue loss disease, biofouling

Abstract

Stony coral tissue loss disease (SCTLD) is a troubling, new disease that is spreading rapidly across the greater Caribbean region, but the etiological agent(s) and the mechanisms(s) of spread are both unknown. First detected off the coast of Miami, major ocean currents alone do not explain the pattern of spread, with outbreaks occurring across geographically disjunct and distant locations. This has raised concerns by researchers and resource managers that commercial vessels may contribute as vectors to spread of the disease. Despite existing regulatory and management strategies intended to limit coastal marine invasion risks, the efficacy of these measures is still unresolved for ship-borne microorganisms, and disease transport via ballast water and hull biofouling are under examination given the high ship traffic in the region. Here, to help inform the discussion of ships as possible vectors of SCTLD, we provide an overview of the current state of knowledge about ships and their potential to transfer organisms in the greater Caribbean, focusing in particular on ballast water, and outline a set of recommendations for future research.

# Introduction

Stony coral tissue loss disease (SCTLD), a lethal coral disease first reported off the coast of Miami-Dade County, Florida in September 2014 (Precht et al., 2016; Walton et al., 2018) has spread quickly, and as of July 2021 has been confirmed in 15 countries/territories throughout the greater Caribbean region (**Fig. 1**; Kramer et al., 2019). Communities depend on coral reef ecosystems for fisheries, coastal protection, and income from tourism. The quick and persistent spread of SCTLD, and still-mysterious mechanism(s) of transport, remain a source of great concern. Because of the unprecedented (for coral diseases) number of coral species affected (~20), there are also concerns that Pacific corals may be susceptible to the disease, prompting concerns about pathogen movement beyond the Caribbean.

As with many other coral tissue loss diseases (Roder et al., 2014; Gignoux-Wolfsohn and Vollmer, 2015), the etiological agent(s) of SCTLD has not been identified. Transmission can occur through water and coral-to-coral contact, and is reduced by antibiotics (Aeby et al., 2019; Muller et al., 2020), indicating biological agents. Ocean currents have been posited as a mode of spread along the Florida Reef Tract (Aeby et al., 2019; Muller et al., 2020), but are not sufficient to explain the spread across the greater Caribbean, motivating the exploration of other modes of transmission including human-mediated transport. One potential mechanism receiving considerable attention is unintended vessel-related transport. While no definitive connection exists between any transport mode and coral disease outbreaks, it is reasonable to consider vessels as potential vectors of pathogens. As the timing and pattern of SCTLD spread through the Caribbean has been better documented than other Caribbean coral disease to date (AGRRA), this disease provides an unprecedented opportunity to examine spatial patterns of coral disease movement.

Vessels are known to be a significant global vector for the spread of aquatic non-native species in coastal waters (Carlton, 1985; Ruiz et al., 2000a; Hewitt et al., 2009; Bailey et al., 2020). A taxonomically diverse biota is transported on or in vessels via ballast water (BW), biofouling (BF) of BW tank walls and of exterior submerged surfaces, bilges, fish holds, fishing gear, and anchor chains (Fofonoff et al., 2003). The role of vessels in transporting organisms varies as a function of vessel size, design, operations, and transit distances/routes. Commercial cargo vessels, as well as recreational, and fishing vessels can all transport non-native organisms (Ashton et al., 2014; Zabin et al., 2014; Ulman et al., 2019).

Compared to macrobiota, our understanding of microbial transport via vessels remains limited, as most research to date has focused on movement of large and easily identifiable species, especially invertebrates and phytoplankton (Lohan et al., 2020). An abundant and diverse assemblage of microorganisms, including pathogens and parasites, have been found in BW, and in sediments and surface biofilms of BW tank (Ruiz et al., 2000a; Drake et al., 2007; Lohan, 2015). However, the extent to which invasions and disease epizootics result from microorganism transfers remains poorly resolved (Lohan et al., 2020). Further, the transport of microorganisms in vessels’ external BF has been especially poorly studied.

Here, we share our perspective on large commercial vessels (hereafter “ships”), which transport cargo and people, as potential vectors for pathogens causing SCTLD, focusing particularly on BW. We do not consider all means by which ships can transport organisms or recreational vessels. We provide a brief overview of BW and relevant U.S. and international regulations on BW management. We then summarize current efforts to evaluate ships as a potential vector for SCTLD. Finally, we provide recommendations for future research to address key knowledge gaps. We intend this perspective piece to inform research and management strategies on the potential for ship-mediated spread of SCTLD.

## 2 Ballast Water (BW)

## 2.1 Mechanism of Organism Transport

BW is fresh or seawater, not wastewater, held in tanks and holds of ships to manage draft, trim, and stability. BW is taken up both in ports and while in transit, and contains a wide diversity of organisms from the water column and shallow benthic sediments (Ruiz et al., 2000a; Drake et al., 2001; Drake et al., 2007). Rarely, ships obtain BW from municipal water sources (NBIC, 2021). Ships take on and discharge BW at various locations (e.g., in-harbor while undergoing cargo operations, at anchorage, at a lightering area outside of harbor where cargo is transferred between ships, or en route between port locations) resulting in large-scale species transfer (NRC, 2011). For context, in 2018, ships reported discharges of 365.2 million m^3^ of BW into U.S. waters (NBIC, 2021).

While ships are in the water, BW tanks are never completely empty, due to limitations of the pumping systems. Consequently, there can be significant amounts of residual BW and sediment in “empty” tanks (Bailey et al., 2003), and biota from many locations may accumulate in BW tanks over time. Thus, the “source” of BW and its associated organisms cannot be attributed exclusively to the last departure place of the ship, because BW may be taken up and comingled across a variety of locations.

## 2.2 Transport of microbes in ballast tanks

A diverse assemblage of microbes, including bacteria and viruses, is carried in BW (Ruiz et al., 2000a; Drake et al., 2001; Drake et al., 2007; Aguirre-Macedo et al., 2008; Kim et al., 2015; Pagenkopp Lohan et al., 2016, 2017; Lymperopoulou et al., 2017; Hwang et al., 2018). Additionally, bacteria are also present in biofilms on internal surfaces (Drake et al., 2005, 2007) and in sediments at the bottom of BW tanks (Drake et al., 2007). Surface- and sediment-associated microbes may be shed within BW tanks into the resident BW via biological and physical processes, and subsequently discharged with the BW (Drake et al., 2005).

## 2.3 Ballast Water Management

Following several species invasions with large ecological and social impacts in the 1980s (Mills et al., 1993; Oliveira, 2007), BW management regulations have advanced within individual countries and internationally (NRC, 1996, 2011). In the U.S., a series of laws and regulations mandating BW management (**Table 1**) has resulted in a stepwise reduction of coastal organism concentrations discharged in BW over the past 35 years (Minton et al., 2005; Albert et al., 2013; Carney et al., 2017).

Under U.S. Coast Guard (USCG) regulations, all ships that are capable of carrying BW and bound for ports or places in the U.S. are required to submit a BW management report to the National Ballast Information Clearinghouse (NBIC; https:\\www.nbic.si.edu) for each arrival. These reports identify, for every tank of BW discharged, the location and date of all BW sources, discharges, and BW management methods applied.

## 2.3.1 Ballast Water Exchange (BWE)

BWE was originally used by ship masters to reduce the amount of sediment buildup in ballast tanks. This practice was then adopted to reduce transport of coastal organisms. Initial guidelines were disseminated by governments and the International Maritime Organization (IMO) (Locke et al., 1991, p. 47; IMO, 1993, 1997). Under USCG rules, a ship conducting BWE flushes out coastal BW (and organisms therein) from its tanks at least 200 nautical miles (Nm) from any land mass.

BWE is conducted in the open ocean either by continuously pumping three full tank volumes of water through each tank or by pumping out the original water until the pump loses suction, and then refilling. BWE replaces coastal BW (and organisms therein) with open ocean water, reducing the concentration of coastal organisms in BW tanks, but not eliminating them (Minton et al., 2005; Ruiz & Reid, 2007; NRC, 2011). Such reductions in the concentration of coastal organisms are expected to lower the likelihood of invasions, because most BW-associated invasions are reported nearshore (Ruiz et al., 2000a, b) and invasion probability scales with the abundance of organisms released (NRC, 2011).

Under USCG rules, a ship conducts BWE at least 200 nautical miles (Nm) from any land mass. Similar regulations have been established internationally by the IMO (IMO, 2004) under the International Convention for Management of Ships’ Ballast Water and Sediments (2004) (hereafter “the Convention”). Specifically, the Convention requires that ships conduct BWE at least 50 Nm from shore. Ships of participating countries (the U.S. is not) must abide by the Convention’s requirements wherever they operate. Thus, ships of parties to the Convention transiting to non-U.S. places in the greater Caribbean should be conducting BWE >50 Nm from shore whenever possible, if managing BW using BWE. Both U.S. and Convention regulations exempt ships if conducting BWE is deemed unsafe due to ship design or sea conditions, and if BWE would require diversion or delay of voyages. Importantly, USCG requires that exempted ships discharge as little BW as is operationally practicable.

## 2.3.2 Ballast Water Management Systems (BWMS)

To further reduce the number of organisms and invasion risk beyond BWE, USCG and Convention regulations phase in concentration-based limits on organisms in BW discharge. The numeric limits are the same in the two standards: fewer than 10 organisms with a minimum dimension > 50 µm per m^3^; fewer than 10 organisms between 50 and 10 µm per mL; and fewer than 1 colony-forming unit (CFU) of *Vibrio cholerae* (serotypes O1 and O139), 250 CFU of *Escherichia coli*, and 100 CFU of enterococci per 100 mL. These limits are largely achieved through use of a BWMS. Treatment with a BWMS is more effective for meeting concentration-based discharge standards than conducting BWE (Minton et al., 2005; Carney et al., 2017) and does not have the same operational constraints as BWE (i.e., distance from shore or safety).

BWMS are generally comprised of several components, including one or more designed to kill, remove, or render nonviable organisms in BW. Filters are often incorporated to remove larger organisms prior to chemical or physical disinfection. Various chlorine-based oxidants and ultraviolet radiation are the most widely employed disinfecting processes. At the time of writing, the USCG has approved 43 BWMS (<https://cgmix.uscg.mil>) and accepted, for temporary use, over 100 BWMS approved under the Convention.

Besides the human pathogens mentioned above, U.S. and Convention regulations place no limits on general organisms <10 µm. While microbes would presumably be reduced in concentration by BWMS, research is needed on the effects of BWMS on potentially pathogenic microbes. Attention should be paid to marine microbes in a viable but non-culturable state, as current culture-based standards may not reflect actual presence of microbes. Furthermore, concentration limits may not be effective against asexually-reproducing coral pathogens depending on infectious dose and favorability for pathogen reproduction at discharge locations.

**3. Ballast Water and SCTLD**

Appearances of SCTLD at geographically distant locations, and first reports near ports (AGGRA, 2021), has invited questions about BW as a possible vector. In 2019, the U.S. Coral Reef Task Force (https://www.coralreef.gov) asked the U.S. Environmental Protection Agency (EPA) to evaluate BW as a potential SCTLD vector. Subsequently, EPA established a collaboration with USCG and the Smithsonian Environmental Research Center to evaluate BW discharges and shipping patterns within the context of SCTLD spread. Additionally, the Naval Research Laboratory and the Atlantic Oceanographic & Meteorological Laboratory are collaboratively investigating the degree to which SCTLD-exposed water can remain infectious, and the efficacy of UV treatment.

**3.1** **Ballast Water Movement & SCTLD Patterns**

U.S. BW management data and Caribbean-wide SCTLD report information are being used in projects to evaluate BW as a potential vector for SCTLD. However, such analyses are correlative and often do not yet consider other possible mechanisms or interactions. Correlations between disease incidences and ship movements or BW discharge are useful but not sufficient to definitively identify ships as vectors. Importantly, given the extensive maritime traffic in the Caribbean, including both ships and recreational vessels, we urge caution in drawing conclusions based on apparent correlations, in the absence of experiments demonstrating causation.

A preliminary analysis in 2019, including >36,000 BW discharge and management records in the NBIC, spanning Sep 7, 2013 - Aug 6, 2020, detected no clear relationship between BW discharges and SCTLD occurrence in U.S. waters, since sites with SCTLD varied in BW discharge histories, including some with no reported discharge.

Following these initial results, on September 06, 2019, at the request of the U.S. National Oceanic and Atmospheric Administration, USCG, in collaboration with U.S. EPA, issued a Marine Safety Information Bulletin (MSIB; USCG, 2019), advising mariners of the SCTLD outbreak, reminding them of BW management regulations, and recommending voluntary BWE practices to reduce possible spread of the disease. Over the 12 months following the MSIB, the number of ships discharging unmanaged (no measures were taken to reduce the number of organisms prior to discharge) BW within 12 Nm of the US shore on a monthly basis was lower than the average number doing so for the 6 years prior to the MSIB. However, there was not a compensatory increase in the number or proportion of BWE events beyond 50 Nm, as might be expected if ships were following the recommendations in the MSIB, making it impossible to attribute this decrease solely to the MSIB. Instead, the increase in ships using BWMS rather than BWE, combined with the decline in ship arrivals due to the COVID-19 pandemic, may account for some or all of the observed decrease in discharge of unmanaged BW (Everett et al., 2021).

The MSIB, as a rapid communication, avoided the significant administrative timelines associated with new regulations; however, it has not appreciably decreased the potential risk of transporting coral pathogens to U.S. locations in the greater Caribbean through the discharge of unmanaged BW. The increasing use of BWMS to treat BW prior to discharge is expected to significantly reduce the risks of pathogen transport, but efficacy of treatment methods against organisms causing SCTLD needs to be directly tested. If BW is identified as a contributor to SCTLD spread, mitigation may need to include approved treatment systems, and depending on the efficacy of such, new regulations for BW management.

**4. Consideration & Future Research**

SCTLD continues to spread across the greater Caribbean and there are indications that Pacific corals may also be susceptible to the disease (Valerie Paul, personal communication), prompting concern about pathogen movement via ships to other geographic regions. It is therefore imperative that we understand whether the disease can be transferred via ship BW and/or BF, and under what circumstances. While SCTLD warrants such attention due to its significant impacts, it also provides an important model to understand the broader risk associated with microorganism transfers.

While ships clearly have the potential to spread pathogens, understanding the likelihood of ship-mediated spread of SCTLD remains challenging given the unknown etiology of the disease. While successful treatment with antibiotics suggests the pathogen(s) may be bacterial (Aeby, 2019; Neely, 2020), we cannot rule out a possible role of free-living or bacteria-infecting viruses, other microorganisms (Rosales et al., 2020), or water quality (Miller et al., 2016; Cunning et al., 2019; Rosales et al., 2019). Additionally, the potential that SCTLD is caused by a consortium of pathogens complicates development of targeted tests or treatments. Experiments are needed to evaluate the effects of voyage characteristics on transmissibility of SCTLD and treatment with BWMS. Current treatment experiments focus on UV irradiation; however, other disinfection processes widely used in BWMS should also be investigated. Ultimately, identifying the pathogen(s) will greatly aid effective prevention.

Concerns about ship-borne transport of SCTLD have focused on free-living pathogens in BW, but pathogens could also be spread as biofilms on internal or external surfaces, or within animal hosts. Zooplankton, commonly transported in BW, can act as a vector for other coral diseases (Certner, 2017). In addition, animals such as snails (Gignoux-Wolfsohn, 2012; Nicolet, 2013) and fireworms (Sussman, 2003) have been shown to harbor and transmit coral pathogens. Understanding the ecology of SCTLD, including potential biotic reservoirs and vectors in BW and BF, is crucial to controlling disease spread via ships. As with BW, the flux of BF organisms is potentially large, given the cumulative surface area that arrives from other bioregions to U.S. waters alone (Miller et al., 2018), and the still limited requirements for BF management (Davidson et al., 2016; Davidson et al., 2018; Tamburri et al., 2020, Georgiades et al. 2021). Furthermore, numerous smaller vessels (e.g., recreational, fishing, and barges) also may transfer biofouling organisms across the greater Caribbean region. Currently, we know of no broadly applicable national or international biosecurity requirements regarding BF in the greater Caribbean region.

There is a demonstrated link between temperature and SCTLD, with lesion progression slowing during periods of thermal stress (Meiling, 2020). Investigation of correlations between SCTLD outbreaks and ships should consider possible interactions with environmental conditions, especially in the context of climate and habitat changes. Next steps should include a statistically-rigorous spatio-temporal analysis of the relationships between SCTLD occurrence, currents, environmental covariates (temperature, salinity, turbidity), and ship/BW flux. This analysis should include the alternate hypothesis that spatio-temporal patterns of outbreaks are not due to pathogen transport, but rather a trigger (e.g., environmental change) inducing pathogenicity. BW information in the NBIC provides a rich data source on ships and BW discharges in U.S. waters. Similar information for non-U.S. ports in the greater Caribbean region would be a valuable addition to this analysis.

Importantly, safety and route exemptions from requirements for BWE (considered necessary for ship safety and smooth maritime transportation) mean that a significant proportion of BW flux among ports within the region will remain unmanaged until existing ships operating in the region reach their U.S and Convention discharge standard compliance dates. Additional interim best management practices, and/or emergency measures, to reduce the transport of SCTLD by these otherwise exempted ships should be considered.

While efforts are underway to understand the potential pathways by which SCTLD spreads geographically, no definitive connection between any transport mode and disease outbreaks has yet been identified. It is reasonable to recognize ships as potential vectors for transmission of SCTLD, via BW and BF, at a large geographic scale; however, their roles in the dynamics of this disease are still not clear. We recommend continued focused research on these possible mechanisms of spread, in concert with ongoing efforts to understand disease etiology and ecology. We also encourage collaborations that bring together experts in ships/shipping, water disinfection, and coral disease.

# Conflict of Interest

The authors declare that the development of this perspective was conducted in the absence of any commercial or financial relationships that could be construed as a potential conflict of interest.

# Author Contributions

All authors were involved in writing the manuscript.

# References

Aeby, G. S., Ushijima, B., Campbell, J. E., Jones, S., Williams, G., Meyer, J. L., et al. (2019). Pathogenesis of a tissue disease affecting multiple species of corals along the Florida reef tract. Front. Mar. Sci. 6: 678. doi: 10.3389/fmars.2019

Albert R.J., Lishman J.M., Saxena J.R. (2013). Ballast water regulations and the move toward concentration-based numeric discharge limits. Ecol. Appl. 23:2, 289-300. doi: 10.1890/12-0669.1. PMID: 23634581

[AGGRA] Atlantic Gulf Rapid Reef Assessment, (2020). Stony Coral Tissue Loss Disease (SCTLD) Outbreak in Caribbean, www.agrra.org, (accessed March 16, 2021).

Ashton G.V., Davidson I.C., and Ruiz, G.M. (2014). Transient small boats as a long-distance coastal vector for dispersal of biofouling organisms. Estuar. Coast. 37, 1572–1581. doi: 10.1007/s12237-014-9782-9

Bailey, S.A., Duggan, I.C., van Overdijk, C.D.A., Jenkins, P.T., and MacIsaac, H.J. (2003). Viability of invertebrate diapausing eggs collected from residual ballast sediment. Limnol. Oceanogr. 48, 1701-1710. doi: 10.1111/j.1365-2427.2004.01185.x

Bailey S.A., Brown L., Campbell, M.L., Carlton, J.T., Canning-Clode, J., Castro, N., et al., (2020). Trends in the detection of aquatic non-indigenous species across global marine, estuarine, and freshwater ecosystems: a 50-year perspective. Divers. Distrib. 26: 1780–1797. doi: https://doi.org/10.1111/ddi.13167

Carlton, J. T. (1985). Transoceanic and interoceanic dispersal of coastal marine organisms: the biology of ballast water. Oceanog. Mar. Biol., 23: 313–371.

Carney, K., Minton, M., Holzer, K., Miller, A.W., McCann, L., and Ruiz, G. (2017). Evaluating the combined effects of management and trade dynamics on transfers of marine organisms by ships’ ballast water. PLoS ONE 12: 1–20. http://dx.doi.org/10.1371/journal.pone.0172468.

Certner, R.H., Dwyer, A.M., Patterson, M.R., and Vollmer, S.V. (2017). Zooplankton as a potential vector for white band disease transmission in the endangered coral, *Acropora cervicornis*. PeerJ 5: e3502. <https://doi.org/10.7717/peerj.3502>.

Cunning R., Silverstein R. N., Barnes B. B., Baker A. C. (2019). Extensive coral mortality and critical habitat loss following dredging and their association with remotely-sensed sediment plumes. Mar. Pollut. Bull. 145 185–199. 10.1016/j.marpolbul.2019.05.02

Davidson, I., Scianni, C., Hewitt, C., Everett, R., Holm, E., Tamburri, M., and Ruiz, G. (2016). Mini-review: Assessing the drivers of ship biofouling management – aligning industry and biosecurity, Biofouling, 32: 4, 411-428. doi: 10.1080/08927014.2016.1149572

Davidson, I., Scianni, C., Minton, M., and Ruiz, G. (2018). A history of ship specialization and consequences for marine invasions, management, and policy. J. Appl. Ecol. 55: 4, 1799–1811. http://dx.doi.org/10.1111/1365-2664.13114

Drake, L.A., Choi K-H, Ruiz, G.M., and Dobbs F.C. (2001). Global redistribution of bacterioplankton and vibrio plankton communities. Biol. Invasions. 3: 193–199. doi: 10.1023/A:1014561102724.

Drake, L.A., Meyer, A.E., Forsberg, R.L., Baier, R.E., Doblin, M.A., Heinemann, S., Johnson, W.P., Koch, M., Rublee, P.A. & Dobbs, F.C. (2005). Potential invasion of microorganisms and pathogens via ‘interior hull fouling’: biofilms inside ballast water tanks. Biol. Invasions. 7: 969–982. doi: https://doi.org/10.1007/s10530-004-3001-8

Drake, L.A., Doblin, M.A., and Dobbs, F.C. (2007). Potential microbial bioinvasions via ships' ballast water, sediment, and biofilm. Mar. Pollut. Bull., 55(7-9): 333–41. doi: 10.1016/j.marpolbul.2006.11.007.

Fofonoff, P.W., G.M. Ruiz, B. Steves, and J.T. Carlton. (2003). “In ships or on ships? Mechanisms of transfer and invasion for non-native species to the coasts of North America,” in Invasive species: vectors and management strategies, Eds. G.M. Ruiz and J.T. Carlton (Washington, DC: Island Press. 152-182.

Everett, R.A., Gignoux-Wolfsohn, S. A., Miller, A.W., Minton, M.S., Rosenau, N.A., Ruiz, G.M. (2021) Patterns of ballast water management in US waters in the greater Caribbean during the Stony Coral Tissue Loss Disease outbreak (2014 - 2020): analyses of National Ballast Information Clearinghouse data. I. Effect of USCG Marine Safety Information Bulletin. National Ballast Information Clearinghouse February 23, 2021. https://nbic.si.edu/wp-content/uploads/2017/09/MSIB.07.19_SCTLD_analysis.pdf

Georgiades, E., Scianni, AC., Davidson, I., Tamburri, M.N., First, M.R., Ruiz, G. Ellard, K., Marty Deveney, M., and Kluza, D. (2021). The role of vessel biofouling in the translocation of marine pathogens: management considerations and challenges, Front. Mar. Sci., 8:435. doi: 10.3389/fmars.2021.660125

Gignoux-Wolfsohn, S., Marks, C. and Vollmer, S. (2012).White Band Disease transmission in the threatened coral, *Acropora cervicornis*. Sci. Rep. 2, 804. https://doi.org/10.1038/srep00804

Gignoux-Wolfsohn, S. A. and Vollmer, S. V. (2015). Identification of candidate coral pathogens on White Band Disease-infected Staghorn Coral. PloS ONE, 10(8): e0134416. doi:10.1371/journal.pone.0134416

Hewitt C.L., Gollasch S., Minchin D. (2009) The Vessel as a Vector – Biofouling, Ballast Water and Sediments. In: Rilov G., Crooks J.A. (eds) Biological Invasions in Marine Ecosystems. Ecological Studies (Analysis and Synthesis), vol 204. Springer, Berlin, Heidelberg. <https://doi.org/10.1007/978-3-540-79236-9_6>

Hwang, J., Park, S.Y., Lee, S., and Lee, T-K. (2018). High diversity and potential translocation of DNA viruses in ballast water, Mar. Pollut. Bull., 137, 449-455. doi: https://doi.org/10.1016/j.marpolbul.2018.10.053

Kramer, P.R., Roth, L., and Lang, J. (2019). Map of Stony Coral Tissue Loss Disease Outbreak in the Caribbean. www.agrra.org. ArcGIS Online. [accessed March 23, 2021].

International Maritime Organization (1993). Resolution A.774(18) 4 Nov. 1993. Guidelines for preventing the introduction of unwanted aquatic organisms and pathogens from ships’ ballast water and sediment discharges. London, U.K.

International Maritime Organization (1997). Resolution A.868(20) 27 Nov. 1997. Guidelines for the control and management of ships’ ballast water to minimize the transfer of harmful aquatic organisms and pathogens. (replaced A.774(18)). London, U.K.

International Maritime Organization (2004). International Convention for the Control and Management of Ship’s Ballast Water and Sediments (2004). In BWM/CONG/36, London, UK.

Kim, Y., Aw, T.G., Teal, T.K., and Rose, J.B. (2015) Metagenomic investigation of viral communities in ballast water. Environ. Sci. Technol. 49: 8396–8407. doi: 10.1021/acs.est.5b01633

Locke, A., Reid, D. M., Sprules, W. G., Carlton, J. T., and van Leeuwen, H. C. (1991). Effectiveness of mid-ocean exchange in controlling freshwater and coastal zooplankton in ballast water. Canadian Technical Report of Fisheries and Aquatic Sciences, 1822.

Lohan, K.M., Fleischer, R.C., Holzer, K.K., Carney, K.J., and Ruiz, G.M. (2015). Amplicon-based pyrosequencing reveals the diversity of microbial eukaryotes in ships’ ballast water: implications for biogeography and infectious diseases. Microb. Ecol. 71: 530–542. doi: https://doi.org/10.1007/s00248-015-0684-6

Lohan, K.M.P., Ruiz, G.M., and Torchin M.E. (2020). Invasions can alter marine disease dynamics. In: Behringer, Silliman, & Lafferty (editors), Marine Disease Ecology. Oxford University Press. doi:10.1093/oso/9780198821632.003.0007.

Lymperopoulou, Despina & Dobbs, F.C. (2017). Bacterial diversity in ships' ballast water, ballast-water exchange, and implications for ship-mediated dispersal of microorganisms. Environ. Sci. Tchnol.. 51: 1962–1972. doi: 10.1021/acs.est.6b03108.

Miller M. W., Karazsia J., Groves C. E., Griffin S., Moore T., Wilber P., et al. (2016). Detecting sedimentation impacts to coral reefs resulting from dredging the Port of Miami, Florida USA. PeerJ 4:e2711. 10.7717/peerj.2711

Miller, A.W., Davidson, I.C., Minton, Steves, B., Moser, C.S., Drake, L.A. and Ruiz, G.M. (2018). Evaluation of wetted surface area of commercial ships as biofouling habitat flux to the United States. Biol. Invas. 20: 1977–1990.

Mills, E.L., Leach, J.H., Carlton, J.T., and Secor, C.L. (1993). Exotic species in the Great Lakes: a history of biotic crises and anthropogenic introductions. J. Great Lakes Res. 19:1–54. doi: http://dx.doi.org/10.1016/S0380-1330(93)71197-1

Minton, M. S., Verling, E., Miller, A. W., and Ruiz, G.M. (2005). Reducing propagule supply and coastal invasions via ships: effects of emerging strategies. Front. Ecol. Environ. 3: 304-308. doi: https://doi.org/10.1890/1540-9295(2005)003[0304:RPSACI]2.0.CO;2

Muller, E., Sartor, C., Aklcaraz, N., and van Woesik, R. (2020). Spatial epidemiology of the Stony-Coral-Tissue-Loss Disease in Florida. Front. Mar. Sci. 7, 163. doi: 10.3389/fmars.2020.00163

National Ballast Information Clearinghouse (NBIC). (2021). NBIC Online Database. Electronic publication, Smithsonian Environmental Research Center & United States Coast Guard. Available from http://dx.doi.org/10.5479/data.serc.nbic; searched on December 4, 2020.

National Research Council (1996). Stemming the Tide: Controlling Introductions of Nonindigenous Species by Ships' Ballast Water. Washington, DC: The National Academies Press. https://doi.org/10.17226/5294.

National Research Council (2011). Assessing the Relationship Between Propagule Pressure and Invasion Risk in Ballast Water. Washington, DC: The National Academies Press. https://doi.org/10.17226/13184

Neely KL, Macaulay KA, Hower EK, Dobler MA. Effectiveness of topical antibiotics in treating corals affected by Stony Coral Tissue Loss Disease. PeerJ. 2020;8:e9289. Published 2020 Jun 9. doi:10.7717/peerj.9289

Nicolet, K.J, Hoogenboom, M.O., Gardine,r N.M., Pratchett, M.S., Willis B.L. (2013). The corallivorous invertebrate Drupella aids in transmission of brown band disease on the Great Barrier Reef. Coral Reefs 32:585-59.

Oliveira, O.M.P. (2007). The presence of the ctenophore *Mnemiopsis leidyi* in the Oslofjorden and considerations on the initial invasion pathway to the North and Baltic Seas. Aquat. Invasions. 2: 185–189. doi: 10.3391/ai.2007.2.3.5

Pagenkopp Lohan, K. M., Fleischer, R. C., Carney, K. J., Holzer, K. K., & Ruiz, G. M. (2016). Amplicon‐based pyrosequencing reveals the diversity of microbial eukaryotes in ships’ ballast water: Implications for biogeography and infectious diseases. Microb. Ecol. 71: 530–542. doi: 10.1007/s00248-015-0684-6

Pagenkopp Lohan, K.M., Fleischer, R.C., Carney, K.J., Holzer, K.K., and Ruiz, G.M. (2017) Molecular characterization of protistan species and communities in ships’ ballast water across three U.S. coasts. Divers. Distrib. 23: 680–691. https://doi.org/10.1111/ddi.12550

Precht, W. F., Gintert, B. E., Robbart, M. L., Fura, R., and van Woesik, R. (2016). Unprecedented disease-related coral mortality in Southeastern Florida. Sci. Rep. 6, 31374. doi: 10.1038/srep31374.

Roder, C., Arif, C., Daniels, C., Weil, E. and Voolstra, C. (2014). Bacterial profiling of White Plague Disease across corals and oceans indicates a conserved and distinct disease microbiome. Mol. Ecol. 23: 965–974. doi:10.1111/mec.12638

Roth, L., Kramer, P.R., Doyle, E., and O’Sullivan, C. (2020). Caribbean SCTLD Dashboard. www.agrra.org. ArcGIS Online. [Accessed April 28, 2021].

Rosales S. M., Sinigalliano C., Gidley M., Jones P. R., Gramer L. J. (2019). Oceanographic habitat and the coral microbiomes of urban-impacted reefs. PeerJ 7:e7552. 10.7717/peerj.7552

Rosales SM, Clark AS, Huebner LK, Ruzicka RR, Muller EM. (2020). *Rhodobacterales* and *Rhizobiales* Are Associated With Stony Coral Tissue Loss Disease and Its Suspected Sources of Transmission. Front. Microbiol. 11:681. Published 2020 Apr 23. doi:10.3389/fmicb.2020.00681

Ruiz G.M., Rawlings T.K., Dobbs F.C., Drake L.A, Mullady T., Huq, A and Colwell R.R. (2000a) Global spread of microorganisms by ships. Nature, 408: 49–50. doi: 10.1038/35040695

Ruiz, G. M., Fofonoff, P. W., Carlton, J. T., Wonham, M.J., and Hines, A.H. (2000b). Invasion of coastal marine communities in North America: apparent patterns, processes, and biases. Annu. Rev. Ecol. Evol. 31, 481–531.

Ruiz, G. M., and Reid, D. F. (2007). Current state of understanding about the effectiveness of BWE (BWE) in reducing aquatic nonindigenous species (ANS) introductions to the Great Lakes Basin and Chesapeake Bay, USA: synthesis and analysis of existing information. (NOAA Technical Memorandum GLERL-142). Ann Arbor: U.S. Department of Commerce & National Oceanic and Atmospheric Administration.

Sussman, M., Loya, Y., Fine, M., and Rosenberg E. (2003). The marine fireworm Hermodice carunculata is a winter reservoir and spring-summer vector for the coral-bleaching pathogen *Vibrio shiloi*. Environ. Microbiol. 5 (4):250-255. doi: 10.1046/j.1462-2920.2003.00424.x. PMID: 12662172

Tamburri, M.N., Davidson, I.C., First, M.R., Scianni, C., Newcomer, K.A., Inglis, G.J, Georgiades E.T., Barnes J., and Ruiz, G.M. (2020). In-water cleaning and capture to remove ship biofouling: an initial evaluation of efficacy and environmental safety. Front. Mar. Sci. 7:437 doi: 10.3389/fmars.2020.00437

Ulman, A, Ferrario, J, Forcada, A, Seebans, H., Arvanitidis, C., Occhipinti-Ambrogi, A., and Marchini, A (2019). Alien species spreading via biofouling on recreational vessels in the Mediterranean Sea. J. Appl. Ecol. 56: 2620–2629. https://doi.org/10.1111/1365-2664.13502

United States Coast Guard (2019) Ballast Water Best Management Practices to Reduce the Likelihood of Transporting Pathogens That May Spread Stony Coral Tissue Loss Disease. Marine Safety Information Bulletin 07–19, Sept. 6, 2019.

USEPA. Final issuance of National Pollution Discharge Elimination System (NPDES) Vessel General Permit (VGP) for Discharges Incidental to the Normal Operation of Vessels, 2013.

Walton, C. J., Hayes, N. K., and Gilliam, D. S. (2018). Impacts of a regional, multiyear, multi-species coral disease outbreak in Southeast Florida. Front. Mar. Sci. 5, 323. doi: 10.3389/fmars.2018.00323.

Zabin, C.J., Ashton C.V., Brown, C.W, Davidson, I.C, Sytsma, M.D, Ruiz, G.M. (2014). Small boats provide connectivity for non-native marine species between a highly invaded international port and nearby coastal harbors. Management of Biological Invasions 5: 97–112. 14). doi: <http://dx.doi.org/10.3391/mbi.2014.5.2.03>

**Table 1.** Timeline of significant legislation and regulation in the U.S. regarding ballast water management. BWM: ballast water management; BWE: ballast water exchange; USC: U.S. Congress; USCG: U.S. Coast Guard; US EPA: U.S. Environmental Protection Agency; U.S.C: U. S. Code; FR: Federal Register.

| Year | Author | Publication | Action |
| --- | --- | --- | --- |
| 1990 | USC | Law - Nonindigenous Aquatic Nuisance Prevention and Control Act (NANPCA; 16 U.S.C. 4711 et seq.) | Established USCG regulatory jurisdiction over BW management |
| 1993 | USCG | Regulation - Ballast Water Management for Vessels Entering the Great Lakes (58 FR 18330) | Ships required to conduct BWE prior to entry into the Great Lakes |
| 1994 | USCG | Regulation - Ballast Water Management for Vessels Entering the Hudson River (59 FR 67632) | Ships required to conduct BWE prior to entry into the Hudson River |
| 1996 | USC | Law - National Invasive Species Act (NISA; 16 U.S.C. 4711 et seq.) | USCG required to establish national BWM guidelines and reporting requirements, evaluate industry participation, and report to Congress |
| 1999 | USCG | Regulation- Implementation of the National Invasive Species Act of 1996 (64 FR 26672) | Established voluntary BWM guidelines, including BWE; recordkeeping and reporting requirements |
| 2002 | USCG | Report to Congress on the Voluntary National Guidelines for Ballast Water Management  (USCG-2002-13147 at http://www.regulations.gov) | Reported a finding of low participation by industry and poor reporting. Triggered NISA requirement that voluntary program become mandatory |
| 2004 | USCG: | Regulation - Penalties for  Non-submission of Ballast Water Management Reports  (69 FR 32864) | Instituted penalties for failure to submit required reports and maintain required records |
| 2004 | USCG: | Regulation - Mandatory Ballast Water Management Program for U.S. Waters (69 FR 44952) | Ships required to conduct a mid-ocean BWE, retain BW onboard, or use an approved alternative BWM method |
| 2005 | USCG: | Policy - Ballast Water Management for Vessels Entering the Great Lakes that Declare No Ballast Onboard (70 FR 51831) | Established BMP: ships entering the Great Lakes with empty BW tanks should conduct mid-ocean flush of “empty” tanks |
| 2008 | USEPA | Vessel General Permit (VGP; 73 FR 79473)) | Established BWM requirements for ships under the Clean Water Act (CWA): USCG requirements plus BWE for ships engaged in Pacific nearshore voyages, and mid-ocean flush of ‘empty” BW tanks prior to entry into Great Lakes |
| 2012 | USCG | Standards for Living Organisms in Ships’ Ballast Water Discharged in U.S. Waters (77 FR 17254) | Ships required to use approved BWMS to meet BW discharge standard, use public water supplies, or discharge to reception facilities; phase out BWE |
| 2013 | USEPA | VGP (78 FR 21938) | Applied USCG BW discharge standard to a broader suite of vessels than USCG rule |
| 2018 | USC | Vessel Incidental Discharge Act (VIDA; 33 U.S.C. 1322(p)) | Repealed existing USCG and EPA authorizations, effective upon publications of new ship discharge standards by EPA and compliance regulations by USCG |

**Figure 1.** Spatial distribution of Stony Coral Tissue Loss Disease across the Caribbean through time. Map modified from Roth et al. (2020).
